# Sex assignment and psychosexual peculiarities of individuals with different forms of androgen insensitivity syndrome: A qualitative study

**DOI:** 10.18502/ijrm.v21i12.15036

**Published:** 2024-01-25

**Authors:** Jenaro Kristesashvili, Levan Kobaladze, Mariam Chipashvili, Anna Jibladze

**Affiliations:** ^1^Medical Faculty of Tbilisi State University, Tbilisi, Georgia.; ^2^Center for Reproductive Medicine Universe, Tbilisi, Georgia.

**Keywords:** Androgen insensitivity syndrome, Androgen receptor, Sex development disorders, Ambiguous genitalia.

## Abstract

**Background:**

A mismatch between chromosomal, gonadal, and phenotypic sexes in individuals with androgen insensitivity syndrome (AIS) creates problems in sex assignment and psychosexual identification.

**Objective:**

To identify psychosexual and sex assignment peculiarities of individuals with different forms of AIS.

**Materials and Methods:**

In this qualitative study, 41 individuals with AIS aged between 15 and 31 yr who referred to the Universe Center for Reproductive Medicine Tbilisi, Georgia between 2016 and 2021 were studied. All individuals underwent clinical, genealogical, hormonal, ultrasonographic, and cytogenetic examinations. In-depth interviews and medical records assessed psychosexual profiles and sex assignment histories.

**Results:**

32 cases were diagnosed with the complete form of AIS (CAIS), 8 individuals with the partial form (PAIS), and one with a mild form (MAIS). Individuals with CAIS and PAIS were assessed at birth and raised as girls. Individuals with CAIS and female psychosexual disposition were referred to us due to amenorrhea. Adolescent individuals with PAIS assessed as girls referred to us due to masculinization detected in puberty. An individual with MAIS was assessed at birth and raised as a boy with male genitalia. All individuals with AIS had typical hormonal data and sex chromosome complex for men. 20 sexually active individuals with CAIS had penile-vaginal contact with the man. None of the individuals with CAIS and PAIS thought about gender reassignment after being diagnosed, only the individual with MAIS aimed for male-to-female transition.

**Conclusion:**

Psychosexual identification remains a significant challenge in AIS management. Detection of female psychosexual disposition in one participant that is unusual to MAIS may be associated with somatic mosaicism of the androgen receptor gene.

## 1. Introduction

Androgen insensitivity syndrome (AIS) is a rare pathology; characterized by wide heterogeneity of clinical manifestations (1-4). AIS is particularly interesting because of the discrepancy between the genetic, gonadal, and phenotypic sexes, which are expressed to disparate degrees in different forms of AIS. Thus, the description of each case of AIS is extremely important in the identification and diagnosis of phenotypic variability.

It is well known that AIS is an X chromosome-linked recessive disease, and its gene is localized in the long arm of the X chromosome Xq11-q12. The application of molecular-genetic research methods showed that the diversity of mutant genes determines AIS phenotypic variations. More than 1000 mutations causing AIS have already been identified (5-11).

AIS is a group of heterogeneous conditions. Its phenotypic range varies from typical female external genitalia to atypical genitalia with a wide spectrum of undervirilization and typical male external genitalia. 3 main phenotypes are considered: complete (СAIS, absence of sensitivity androgen receptors to androgens), partial (PAIS, decrease of sensitivity androgen receptors to androgens), and mild (MAIS, low degree of impairment of sensitivity of androgen receptors to androgens) androgen insensitivity (1-4).

In cases of CAIS, sex in newborns is unequivocally assessed as feminine. The female phenotype at birth, external genitalia without virilization, and absence of secondary sexual characteristics of men means that such individuals were raised and lived as females for a long period, have female gender and identity, and typical female activity and interests. It is often very difficult for them to perceive that they, phenotypic women, have the male karyotype 46, XY, and testicles (1, 2, 6). In cases of CAIS, the problem arises due to a mismatch between the male sex of the fetus established by antenatal amniocentesis (46, XY) and the newborn with a postnatal female phenotype (1, 2).

The situation is different in cases of PAIS newborns with ambiguous genitalia. In such cases, part of the newborns is evaluated as boys and part as girls, which probably depends on the degree of masculinization of the external genitalia (6, 10, 12). In cases of PAIS, gender reassignment is quite common in individuals' lifetime from childhood, even < 40 yr. Self-initiated gender reassignment occurred in about 9% of cases in individuals assessed as girls and boys at birth (9, 12). Based on literary data analysis, except for rare cases, no gender change is detected in individuals with CAIS (11).

MAIS is characterized by a low degree of insensitivity to androgens. The phenotype of these individuals is male. They have typical men's genitalia. Androgen receptor gene mutations in cases of MAIS are less studied (1, 2, 5, 7).

Usually, such individuals are assessed at birth and then raised as men, with male gender and psychosexual disposition. They visit a doctor due to infertility or gynecomastia detected at puberty. The condition is probably underdiagnosed (1, 2); unlike individuals with CAIS and PAIS whose reproductive prognosis is pessimistic, in some cases of MAIS, fertility might be restored by high doses of androgens. In rare cases, fertility has been reported in individuals with PAIS assessed as males (13).

The study aimed to identify psychosexual sex assignment peculiarities in individuals with different forms of AIS.

## 2. Materials and Methods

In this qualitative study, which was conducted from 2016-2021 at the Universe Center for Reproductive Medicine, Tbilisi, Georgia 41 individuals with AIS were involved. The age of individuals ranged from 15-31 yr. The inclusion criteria was a confirmed diagnosis of AIS. At the beginning of the study for detecting diagnosis of AIS the individuals underwent clinical, gynecological, ultrasound examination (US), hormonal and cytogenetic, and genealogical investigations. Assessment of the sexual development of individuals was performed according to Tanner stages (Ma 0-5, P 0-5, Ax 0-5). Genital organs US examination was conducted by using voluson E10 (General Electric), intravaginal examination for sexually active patients, for virgin patients-abdominal examination. Hormonal levels (prolactin, follicle-stimulating hormone, luteinizing hormone [LH], testosterone, estradiol) were determined by immunoassay method enzyme-linked immunosorbent assay (Beckman Coulter, Irving, TX). A cytogenetic investigation was performed by determining the karyotype in peripheral blood lymphocyte cultures by G-banding. Psychosexual peculiarities and sex assignment history were assessed through in-depth interviews with participants and their parents', medical records were only used if necessary or available. The questions were as follows: were there any problems with sex assignment at birth or later? What types of toys, and clothes have been preferred by the child in childhood-typical for girls or boys? How did He/-She feel in their passport gender? What gender was sexually attractive for the person, etc.

32 individuals were diagnosed with CAIS, 8 with PAIS, and 1 with MAIS. 33 individuals underwent orchiectomy. 25 individuals with CAIS in cases of intraabdominal location of testes underwent orchiectomy to prevent the risk of testicular malignancy. All adolescent individuals with PAIS assessed and raised as girls underwent bilateral orchiectomy, according to the protocol, to stop the progression of masculinization and considering that in the cases of PAIS, the risk of testicular malignancy is increased.

### Ethical considerations

The Ethics Committee of Center for Reproductive Medicine Universe, Tbilisi, Georgia approved the study (Code: 1/1 from 08.01.2016). All the adult participants and parents of adolescent individuals signed a written consent form for reporting their data and were assured about the confidentiality of the information.

## 3. Results

All participants (15-31 yr) of the study applied Universe Center for Reproductive Medicine to define the diagnosis and elaboration of the treatment strategy. The passport sex of all 32 individuals with CAIS and 8 individuals with PAIS was female. Only one individual with MAIS had male passport sex. Due to the specifics of our department (gynecological endocrinology), 32 individuals with CAIS referred to us for amenorrhea, in 8 cases for infertility, and 8 individuals with PAIS with female passport sex and gender-due to masculinization detected at puberty. The referral of an individual with MAIS to our clinic, regardless of the male gender of the passport, was because he considered himself a woman.

Individuals with all forms of AIS studied by us had a female psychosexual disposition. The sex of the infants at birth was assessed as female in cases of CAIS and PAIS, and they were raised as girls. Only one individual with MAIS was assessed as a boy at birth and was raised as a boy.

### CAIS

**Figure 1:**
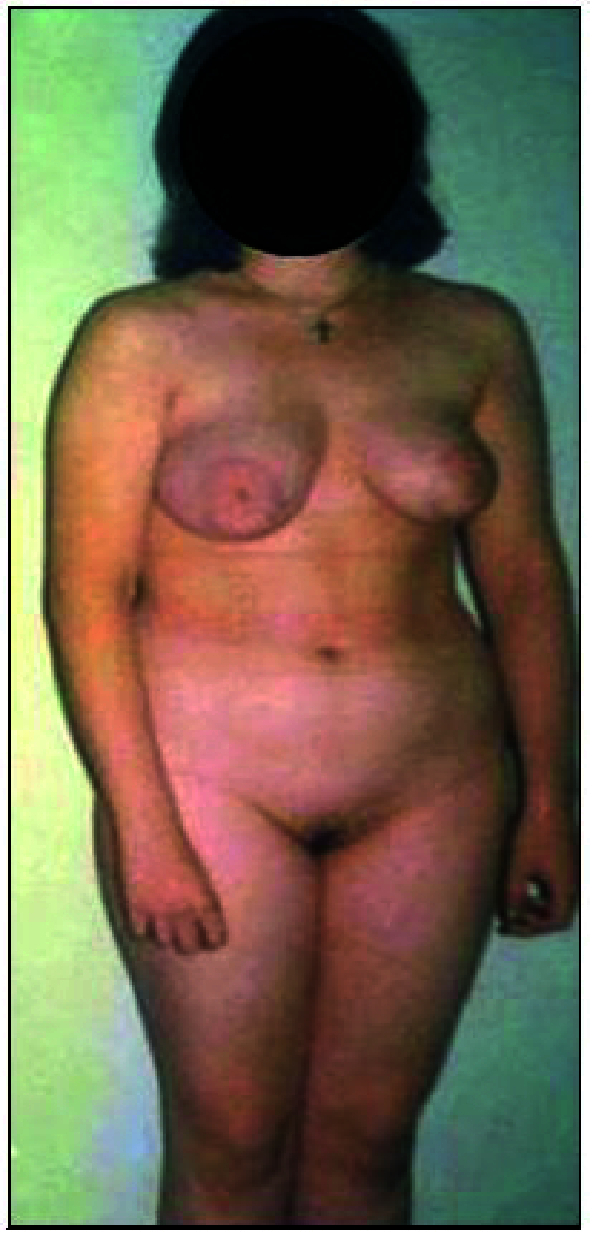
17 yr individual with CAIS.

All individuals with CAIS showed a female appearance with well-developed mamme with slightly pigmented areolae (according to Tanner-Ma_4-5_) (Figure 1). Pubic and axillary hair were not observed in most individuals. Only 6 individuals had scarce pubic hair (according to Tanner-P_1_Ax_0_).

Axillary hair was not observed in any of the individuals with CAIS. Clitoromegaly was also not reported in any of the cases. Labia majora were normally developed and minor ones were hypoplastic. In sexually inactive individuals, the length of the vagina varied within 3-4 cm with the probe, while in sexually active individuals it was elongated to 7-8 cm. The uterus was not detected by palpation and radiological examination. The most common localization of the testicles was unilaterally in the small pelvic cavity above and laterally or with the internal ring of the inguinal canal (13 individuals). In 10 cases, the testicles were bilaterally located in the abdominal cavity. In other cases, the gonads were palpated at the external ring of the inguinal canal or in the thickness of the labia majora.

### PAIS

Individuals with PAIS (n = 8) who were assessed as girls and raised as girls were referred to us at the age of 15-16 yr due to progressive masculinization and absence of menstruation, which was very disturbing for them against the background of female psychosexual disposition. They were concerned with voice deepening, which was not hidden in social relations.

**Figure 2:**
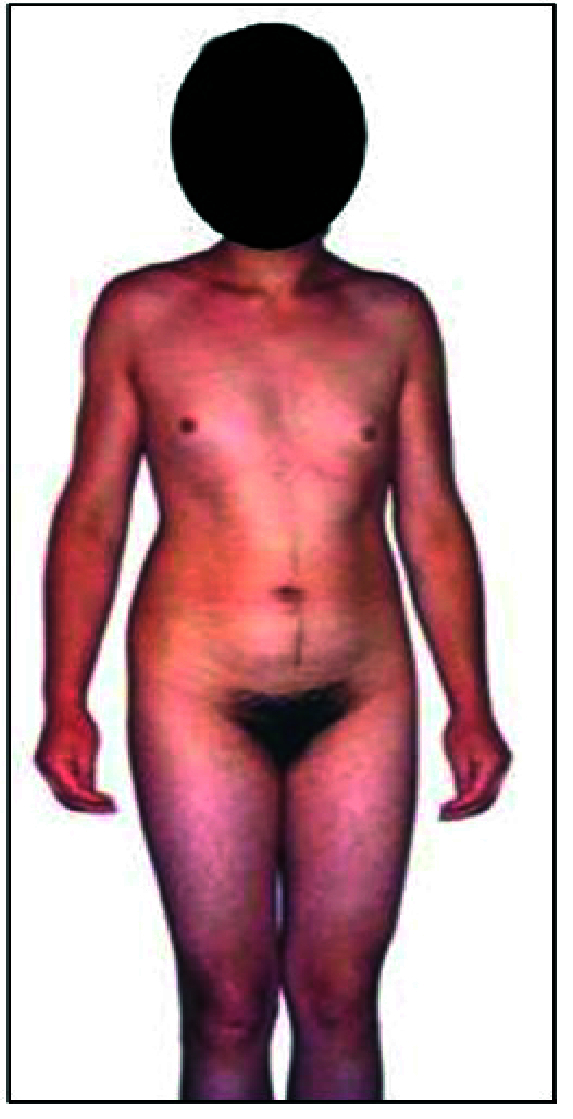
16 yr individual with PAIS.

In contrast to individuals with CAIS, their body composition was masculine, mamme by Tanner varied within the range of Ma_1-3_, pubic and axillary hair varied within P_3-5_, Ax_3-5_. Pubic hair was female type in all cases (Figure 2). Labia majora were normally developed, and minor ones were hypoplastic, Clitoromegaly was observed in all cases (3-4 cm), length of the vagina was 2-4 cm with the probe. Urogenital sinus was found in 2 cases. The uterus was not detected by palpation or visualized by radiological examination in any of the cases. Enlargement of the clitoris, voice deepening, and mamme growth in all cases were reported since the onset of puberty.

Because the presence of urogenital sinus in individuals with PAIS was uncommon, we believe assessing them at birth as girls was important.

Regarding testicular placement, in individuals with PAIS, their placement was more frequent in the external ring of the inguinal canal and the thickness of the labia majora compared to individuals with CAIS (6 individuals).

Cytogenetic examination of all individuals with CAIS and PAIS revealed normal male karyotype 46, XY. Only 1 patient with MAIS showed balanced Robertsonian translocation on the background of the presence of male sex chromosomes-karyotype 46, XY rob (13q14q), which is not substantially related to the underlying disease. Hormonal investigations in all our study participants with AIS detected normal men's testosterone and estradiol levels and infrequently elevated (5 individuals) testosterone levels. Elevated LH levels were found to be more frequent (26 individuals).

Of the 32 studied individuals with CAIS, 12 were married to men, 8 had unmarried sexual relations with men, and in all cases, had no problem establishing penile-vaginal contact. In the background of such sexual contact, vaginal elongation was observed in these individuals, which was sufficient for normal sexual contact. 9 of them had an orgasm. As women, they were satisfied with the quality of their psycho-social and sexual lives.

We observed that none of the individuals with PAIS were sexually active, although they were concerned because they wanted their appearance and voice to be more feminine. The psychosexual disposition of all of them was unwavering, distinctly female. Since childhood, they had been wearing women's clothes, playing with girls' toys dolls, making friends with girls, and imagining boys as the objects of romantic-love relationships in adolescence.

The reaction of individuals with AIS after diagnosis was different, which was more or less severely stressful for all of them. It was equally difficult for them to realize that their reproductive prognosis was pessimistic and that it was impossible to achieve fertility even with the help of assisted reproductive technologies. For one individual with CAIS, being diagnosed at the age of 20 was so shocking that she did not visit us for the next 9 yr and ignored all the recommendations. She showed up at 29 because she was bothered by the formation in the thickness of the labia majora. It turned out she had been married to a man for 7 yr, raising his children from his first marriage, but because of vaginismus, she did not have penil-vaginal sexual contact with her husband. The participant underwent orchiectomy, vaginismus treatment, and was prescribed replacement therapy with estrogen, after which she was satisfied with her sexual life.

All other individuals with CAIS continued to live in the female gender and had no idea of gender reassignment even though they knew they had male genetic sex 46, XY karyotype, and testes.

These individuals have explained the appropriateness of orchiectomy and estrogen replacement therapy after the completion of puberty. 25 individuals with CAIS underwent orchiectomy and were prescribed estrogen replacement therapy.

One 28-yr-old individual with CAIS postponed the orchiectomy because she had good sexual penile-vaginal contact with a man, with frequent orgasms, and feared that the orchiectomy would negatively affect her sexual life.

Individuals with PAIS who grew up as girls had the female psychosexual disposition and, conversely, suffered from the appearance of signs of masculinization and did not think about gender reassignment. All adolescent individuals with PAIS assessed and raised as girls underwent bilateral orchiectomy according to the protocol to stop the progression of masculinization. In the case of PAIS, the risk of testicular malignancy was increased. These individuals underwent feminizing genitoplasty and were prescribed estrogen replacement therapy. After 1-yr follow-up, an increase in mamme, change in tone of voice, and an improvement in mood were observed in these individuals.

In one case, an adolescent with PAIS who was a successful athlete, after learning of the diagnosis and considering that it might pose problems with participating in women's sports competitions, was shocked and considered going to the monastery.

None of the examined CAIS and PAIS individuals, who had been diagnosed with the presence of testicles in childhood as a result of unilateral or bilateral hernia resection, thought about sex reassignment because initial sex, raising sex, and gender sex were female.

Among the examined individuals, 5 familial cases were identified. All cases were found in the families of individuals with CAIS. In 4 cases, CAIS was detected in siblings, which confirmed the CAIS diagnosis. In 2 cases, “aunts" of individuals with CAIS had primary amenorrhea and infertility. Unfortunately, their examination was impossible, although it is likely that they could be considered individuals with CAIS.

No familial cases were detected among individuals with PAIS, and there were no problems with sex assignment among relatives.

### MAIS

As only one participant among the individuals we examined had MAIS and in an atypical form, we considered it appropriate to describe it separately.

A 22-yr-old individual referred due to uncertainty about gender, whereas, against the background of clearly manifested female psychosexual disposition, the individual had typical male external genitalia.

**Figure 3:**
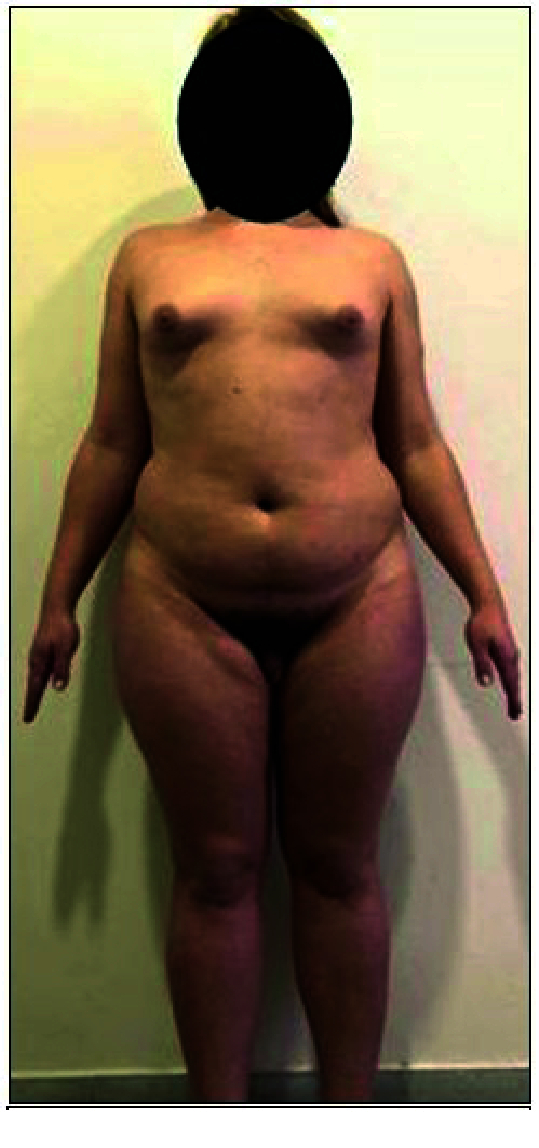
22 yr individual with MAIS.

Objectively height-166 cm, body weight-85 kg, body mass index-31, shoulder circumference-115 cm, pelvic circumference-114 cm, and subcutaneous adipose tissue well expressed and evenly distributed. A complete assessment of hirsutism has not been possible since he was doing epilation. Sexual development according to Tanner-Ma ${\;}_{3}$ P ${\;}_{5}$ Ax ${\;}_{5}$ (Figure 3).

**Figure 4:**
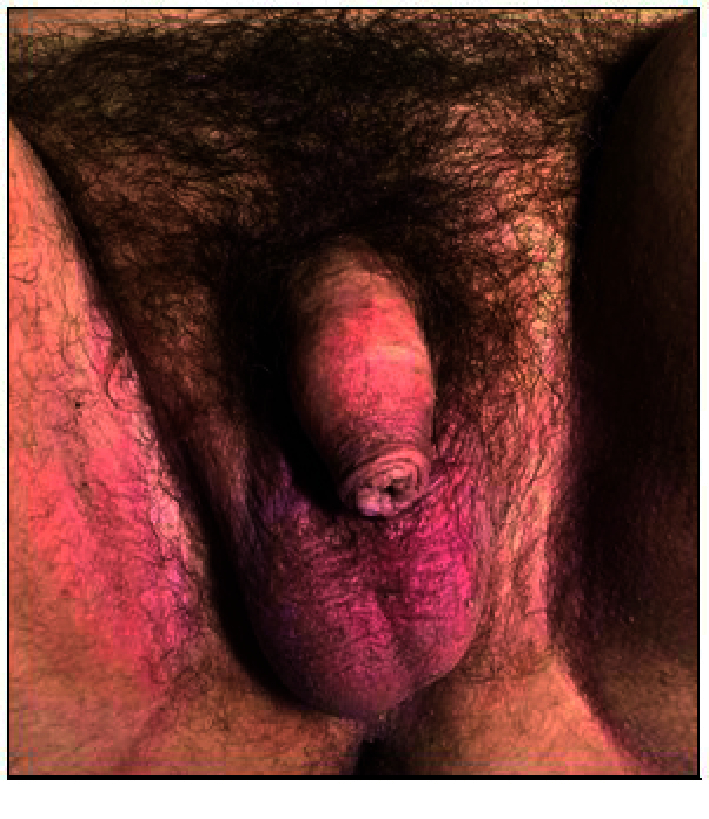
Extrenal genitalia of individual with MAIS.

Pubic hair of female type. External genitalia-male type, length of the penis-5 cm, width-2.5 cm in non-erect position, external urethral orifice opens at the tip of the penis, scrotum wrinkled, pigmented, testicles are present on both sides (Figure 4).

At birth, the newborn was assessed as a boy and later was raised in a family as a boy, although from childhood, he wanted to wear girls' dresses and play with toys typical for girls. From the age of 13, after mamme began to grow, he protested against rearried evaluated sex, and as he got older, the inevitability of being recognized as a woman was reinforced. He wore women's clothing and posed as a woman in newly acquired acquaintances with a woman's name, though the gender on the ID card remained male. Before contacting us, the individual had not undergone any examination.

According to hormonal research, testosterone, estradiol, follicle-stimulating hormone, and LH levels were within the male norm.

According to ultrasonography, the prostate gland, seminal vesicles, epididymal heads, right testicle size, volume-N, and left testis was hypoplastic. The structure of both testicles was homogeneous, echogenicity-N. Cavernous and spongy bodies of the penis-homogeneous. Karyotype of patient-46, XY rob (13q14q).

The participant's psychosexual disposition was female, and she showed interest in men. Individual dreams and plans to feminize external genitalia and create a neovagina by plastic surgery, as a result of which he will be able to change the passport sex to female and live in this sex officially, in non-degrading conditions in which he has imagined himself.

## 4. Discussion

Psychosexual identification is one of the most important challenges in AIS management, as this disorder shows a mismatch between the chromosomal, gonadal, and phenotypic sexes (1-4, 6). 

No problem with sex assignment was observed in individuals with CAIS we studied. Psychosexual disposition and orientation were distinctly female. Sexually active individuals had no problem with penile-vaginal contact, vaginal elongation was observed from 3-4 cm to 7-8 cm, and orgasm was observed in 9 cases.

Such an approach that individuals with CAIS should be raised as women is justified because, in addition to the development of the external genitalia, complete insensitivities to androgens were also reflected in the brain. Our data also confirms this approach. Even though it was quite stressful for our individuals with CAIS to understand that they had a male 46, XY karyotype and testes against a background of female psychosexual disposition and orientation, none of them thought about gender reassignment. They continued to live as women and were satisfied with their lives.

Certain problems develop in individuals with CAIS after being informed of the diagnosis. It is especially difficult for AIS individuals to understand mismatch, be they girls or women with a female phenotype and have a male karyotype and testes. They perceived themselves as stigmatized and developed depression. However, most individuals with CAIS who were raised as girls and lived as women believe this is the best solution for them (1, 9, 12, 14).

It is not excluded that orchiectomy in individuals with CAIS may have a psychological effect. One of our 28-yr-old sexually active participants with CAIS, who had a good sexual life postponed the orchiectomy, fearing that it would worsen the quality of sexual life.

Other sexually active individuals under our observation did not report a change in sexual desire or sexual satisfaction after the orchiectomy.

Interestingly, some individuals with CAIS who underwent orchiectomy and received estrogen replacement therapy complain of gender well-being and sexual dissatisfaction. Some of them reported that decreased libido significantly decreases the frequency and intensity of orgasm after gonadectomy. Some authors suggest that considering the effects of orgasmic function on sexual well-being, androgen therapy may be preferred over estrogen replacement therapy (2). However, this is difficult to explain in CAIS cases in conditions of complete insensitivity to androgens.

The situation is different from the assigned gender in cases of PAIS. Individuals with PAIS under our observation, who were raised as girls, had a female psychosexual disposition. However, male body composition, imperfect breast development, deepening of the voice, and absence of menstruation were identified at puberty. The increasing clitoral virilization has drawn less attention from their parents. The abovementioned complaints were the reason for their referral to the gynecological-endocrine department of our clinic. It should be mentioned that adolescent individuals in this group expressed satisfaction with improving the degree of feminization and well-being in the background of estrogen replacement therapy after orchiectomy. Our participants with PAIS were sexually inactive, and none of them thought about gender reassignment after being diagnosed.

About one-third of individuals with PAIS were dissatisfied with their assigned gender, which becomes the basis for subsequent gender reassignment. However, it is interesting to note that in the case of the discovery of testicles inguinal during childhood, it is on doctors advice that in individuals with PAIS assessed as girls, the female sex is changed to male. Often in adulthood, such individuals return to the female sex by self-initiative (9, 12, 14).

Analysis of the cases described in the literature shows that individuals with PAIS assessed as boys are more likely to experience gender problems during their lifetime, change sex several times, etc., while individuals with PAIS assessed as girls are less likely to face such problems (9, 14).

In turn, this is facilitated by the fact that according to existing protocols, in individuals with PAIS assessed as girls, in case of reduced masculinization intensity at puberty and taking into consideration the higher risk of testicular malignancy in PAIS cases, compared to CAIS cases, orchiectomy is performed in childhood or early puberty, followed by estrogen therapy (15). In cases of PAIS assessed in girls, it may be justified to prescribe combined androgen-estrogen replacement therapy to improve sexual function in the presence of some sensitivity to androgens (16).

Very interesting and unusual was the presence of marked female psychosexual disposition and orientation in a male individual with MAIS, under our observation, against the presence of typically male genitalia. According to some authors initially assigned sex and reared sex led to the formation of gender, which, in the case of this individual, was male, and yet, in the presence of mild androgen insensitivity, female psychosexual disposition was detected (1, 2, 9, 12).

Differences in phenotype manifestations described in the literature in cases of identical androgen receptor mutations, including between members of the same family, indicate the possibility of modifying the effects of other genes and their ability to interact with environmental factors (1, 11, 14). It is also important to consider the presence of somatic mosaicism of androgen receptors in such cases (2, 8).

Despite typical male external genitalia, female psychosexual disposition, and orientation from childhood in a participant with MAIS can be explained by somatic mosaicism of androgen receptors. In this case, the fact that the individual had a male social gender and was raised in the family as a boy should also be considered. Various authors believe that initially assigned sex is important in sexual orientation (9, 12, 14). It is also believed that the more masculinized the genitalia are, the more likely virilization of the brain is (10, 12, 16), which in the case of our participant, did not affect the formation of female psychosexual disposition.

## 5. Conclusion

Psychosexual identification remains a significant challenge in AIS management. Detection in our patient female psychosexual disposition that is unusual to MAIS may be associated with somatic mosaicism of androgen receptor gene.

##  Conflict of Interest

The authors declare that there is no conflict of interest.

## References

[bib1] Batista RL, Frade Costa EM, de Santi Rodrigues A, Lisboa Gomes N, Faria Jr JA, Nishi MY, et al (2018). Androgen insensitivity syndrome: A review. Arch Endocrinol Metab.

[bib2] Ovidiu B, Marcu DR, Dan LD, Mischianu DL, Catalina Poiana C, Camelia C, et al (2022). The challenges of androgen insensitivity syndrome. Arch Med Sci.

[bib3] Jiang X, Teng Y, Chen X, Liang N, Li Z, Liang D, et al (2020). Six novel mutation analysis of the androgen receptor gene in 17 Chinese individuals with androgen insensitivity syndrome. Clin Chim Acta.

[bib4] Liu Q, Yin X, Li P (2020). Clinical, hormonal and genetic characteristics of androgen insensitivity syndrome in 39 Chinese individuals. Reprod Biol Endocrinol.

[bib5] Gottlieb B, Beitel LK, Nadarajah A, Paliouras M, Trifiro M (2012). The androgen receptor gene mutations database: 2012 update. Hum Mutat.

[bib6] Gulia C, Baldassarra S, Zangari A, Briganti V, Gigli S, Gaffi M, et al (2018). Androgen insensitivity syndrome. Eur Rev Med Pharmacol Sci.

[bib7] Eisermann K, Wang D, Jing Y, Pascal LE, Wang Z (2013). Androgen receptor gene mutation, rearrangement, polymorphism. Transl Androl Urol.

[bib8] Batista RL, de Santi Rodrigues A, Machado AZ, Nishi MY, Cunha FS, Silva RB, et al (2018). Partial androgen insensitivity syndrome due to somatic mosaicism of the androgen receptor. J Pediatr Endocrinol Metab.

[bib9] Fisher AD, Ristori J, Fanni E, Castellini G, Forti G, Maggi M (2016). Gender identity, gender assignment and reassignment in individuals with disorders of sex development: A major of dilemma. J Endocrinol Invest.

[bib10] Lek N, Tadokoro-Cuccaro R, Whitchurch JB, Mazumder B, Miles H, Prentice P, et al (2018). Predicting puberty in partial androgen insensitivity syndrome: Use of clinical and functional androgen receptor indices. EBioMedicine.

[bib11] de la Vega JAB, Fernández-Cancio M, Bernal S, Audí L (2015). Complete androgen insensitivity syndrome associated with male gender identity or female precocious puberty in the same family. Sex Dev.

[bib12] Markosyan R, Ahmed SF (2017). Sex assignment in conditions affecting sex development. J Clin Res Pediatr Endocrinol.

[bib13] Tordjman KM, Yaron M, Berkovitz A, Botchan A, Sultan C, Lumbroso S (2013). Fertility after high-dose testosterone and intracytoplasmic sperm injection in a patient with androgen insensitivity syndrome with a previously unreported androgen receptor mutation. Andrologia.

[bib14] Mendonca BB (2014). Gender assignment in individuals with disorder of sex development. Curr Opin Endocrinol Diabetes Obes.

[bib15] Cools M, Looijenga LH, Wolffenbuttel KP, T’Sjoen G (2014). Managing the risk of germ cell tumourigenesis in disorders of sex development individuals. Endocr Dev.

[bib16] Van Hemmen J, Veltman DJ, Hoekzema E, Cohen-Kettenis PT, Dessens AB, Bakker J (2016). Neural activation during mental rotation in complete androgen insensitivity syndrome: The influence of sex hormones and sex chromosomes. Cereb Cortex.

